# Thyroid sequelae of COVID-19: a systematic review of reviews

**DOI:** 10.1007/s11154-021-09653-1

**Published:** 2021-04-11

**Authors:** Pierpaolo Trimboli, Chiara Camponovo, Lorenzo Scappaticcio, Giuseppe Bellastella, Arnoldo Piccardo, Mario Rotondi

**Affiliations:** 1 Clinic for Endocrinology and Diabetology, Lugano Regional Hospital, Ente Ospedaliero Cantonale, Lugano, Switzerland; 2 Faculty of Biomedical Sciences, Università della Svizzera Italiana (USI), Lugano, Switzerland; 3 Division of Endocrinology and Metabolic Diseases, University Hospital “Luigi Vanvitelli”, University of Campania “L. Vanvitelli”, Naples, Italy; 4 Department of Nuclear Medicine, Galliera Hospital, Genoa, Italy; 5 Unit of Internal Medicine and Endocrinology, Laboratory for Endocrine Disruptors, Istituti Clinici Scientifici Maugeri IRCCS, Pavia, Italy; 6 Department of Internal Medicine and Therapeutics, University of Pavia, Pavia, Italy

**Keywords:** Thyroid, COVID-19, Coronavirus, Thyroiditis

## Abstract

The coronavirus disease 2019 (COVID-19) caused by the severe acute respiratory syndrome coronavirus 2 (SARS-CoV-2) has the potential to cause multi-organ effects including endocrine disorders. The impact of COVID-19 on the thyroid gland has been described but several aspects have to be clarified. The systematic review was conceived to achieve more solid information about: 1) which thyroid disease or dysfunction should be expected in COVID-19 patients; 2) whether thyroid patients have a higher risk of SARS-CoV-2 infection; 3) whether the management has to be adapted in thyroid patient when infected. The literature was searched by two authors independently. A 5-step search strategy was a priori adopted. Only reviews focused on the relationship between thyroid and COVID-19 were included. The last search was performed on February 21^st^ 2021. Two-hundred-forty-seven records was initially found and nine reviews were finally included. The reviews identified several potential thyroid consequences in COVID-19 patients, such as thyrotoxicosis, low-T3 syndrome and subacute thyroiditis, while no relevant data were found regarding the potential impact of COVID-19 on the management of patients on thyroid treatment. The present systematic review of reviews found that: 1) patients diagnosed with COVID-19 can develop thyroid dysfunction, frequently non-thyroidal illness syndrome when hospitalized in intensive care unit, 2) having a thyroid disease does not increase the risk for SARS-CoV-2 infection, 3) thyroid patients do not need a COVID-19-adapted follow-up. Anyway, several factors, such as critical illness and medications, could affect thyroid laboratory tests.

## Introduction

In March 2020, WHO declared the pandemic of the novel coronavirus disease 2019 (COVID-19) caused by the severe acute respiratory syndrome coronavirus 2 (SARS-CoV-2) [1]. Since then, about 111 million cases and 2.5 million deaths from SARS-CoV-2 were reported until February 2021 [2]. What has been shocking for the whole medical scientific community is that SARS-CoV-2 has a wide spectrum of clinical severity, ranging from a- or pauci-symptomatic presentation to disease-specific mortality [3]. COVID-19 has the potential to cause upper respiratory tract, pulmonary and systemic inflammation, determining multi-organ dysfunction especially in frail patients [4]. These latter are also at higher risk of COVID-19 specific death. Among the various clinical effects of SARS-CoV-2, the endocrine ones have been investigated. Importantly, diabetic patients were proven to be at higher risk for infection and poorer prognosis when infected [5]. Moreover, treatment modifications are required as a consequence of COVID-19 in patients affected by endocrine diseases, such as diabetes, adrenal insufficiency, hypo- or hyper-natraemia [5, 6]. In addition, the impact of COVID-19 on thyroid gland has been described by direct or indirect effect [7].

In the thyroid field many original papers, several case reports, and some recommendations by endocrine societies on the impact of COVID-19 have been published. Furthermore, a not negligible number of narrative and systematic review articles on this topic have been conducted. Overall, although several clues would suggest a possible relationship between thyroid gland and SARS-CoV-2, and even if the presence of the receptor for SARS-CoV-2 entry (i.e., angiotensin-converting enzyme 2 [ACE-2]) in thyroid cells has been proven [8, 9], which thyroid consequences can be observed in patients diagnosed with COVID-19 remains to be fully clarified.

The present study was conceived to provide more solid information about three specific items: 1) which thyroid disease or dysfunction can be expected in COVID-19 patients; 2) whether thyroid patients have a higher risk of SARS-CoV-2 infection; 3) whether the management has to be adapted in thyroid patient when infected by SARS-CoV-2. Therefore, it was designed a systematic review of reviews published on this topic, the conclusions of the retrieved articles were summarized, and the summary of findings discussed.

## Materials and methods

### Review conduction

The systematic review was conducted according to the methodology proposed by Aromataris et al. [10].

### Search strategy

The literature was searched by two authors independently (PT and LS). A 5-step search strategy was a priori adopted: 1) sentinel studies were sought in PubMed using multiple combinations of the following keywords: thyroid, SARS-CoV-2, coronavirus, COVID, COVID-19; 2) keywords and MeSH terms were identified in PubMed; 3) PubMed, CENTRAL, Scopus, and Web of Science were searched; 4) narrative reviews, systematic reviews and meta-analyses with potential to be eligible were identified; 5) reviews focused only on the relationship between thyroid and COVID-19 were directly included in the study while those focusing on the general endocrine impact by COVID-19 were screened and included only when having a large section dedicated to thyroid. The last search was performed on February 21^st^ 2021. To find additional studies and expand the search, the references of the articles retrieved were also screened.

### Data extraction

For each included article the following information was extracted by two authors independently (CC and LS): authors, country, date of the last literature search, date of publication, journal, type of review (i.e., narrative review, systematic review, systematic review with meta-analysis), context of the review (i.e., thyroid field or endocrinology), aim of the review, conclusions of the authors. Also, the type of thyroid sequelae (i.e., diseases or dysfunction) were extracted.

## Results

### Reviews retrieved

**Fig. 1:**
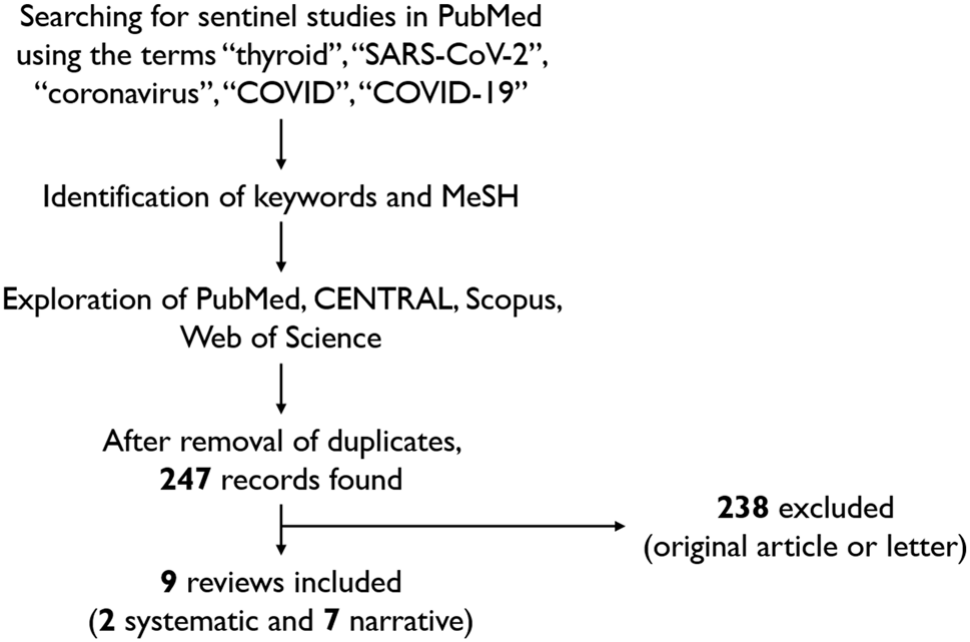
Search strategy and flow of articles

By the search strategy, a number of 247 records was initially found. Among these, according to the above criteria, 238 studies were excluded since they were not review articles, and nine reviews were finally included [7, 11–18] (Fig. 1).

The nine included reviews were conducted by European, American and Asian authors. The date of the last literature search was reported in three studies and varied from September to December 2020. Seven studies were narrative review and two were systematic review. Both objectives and conclusions of the nine reviews were clearly reported. However, since the largest part of the retrieved reviews was narrative, their pre-defined patient-centered questions (e.g., PICOS, participants, interventions, comparators, outcomes, and study design) were not reported. Table 1 summarizes the main features of the reviews included in the present systematic review.

**Table 1:** Main characteristics of the reviews included in the present systematic review

First author [ref]	Country	Last literature search	Date of publication	Journal	Type of review	Topic (thyroid/endocrinology)
Dworakowska [11]	Poland, UK	N.A	June 7th 2020	Endocrine	NR	Thyroid
Gorini [12]	Italy	N.A	September 11th 2020	International Journal of Environmental Research and Public Health	NR	Thyroid
Caron [13]	France	N.A	September 18th 2020	Annales d’endocrinologie	NR	Thyroid
Scappaticcio [7]	Italy, Switzerland, Argentina	September 5th, 2020	November 25th 2020	Reviews in Endocrine and Metabolic Disorders	SR	Thyroid
Kumari [14]	India	N.A	December 10th 2020	Heliyon	NR	Thyroid
Chen [15]	China	December 23th 2020	January 11th 2021	Endocrinology	NR	Thyroid
Speer [16]	Hungary	N.A	January 19th 2021	Endocrine Journal	NR	Thyroid
Croce [17]	Italy	N.A	February 13th 2021	Journal of Endocrinological Investigation	NR	Thyroid
Piticchio [18]	Italy	November 18th 2020	February 13th 2021	Journal of Endocrinological Investigation	SR	Endocrinology

*N.A*. not available, *NR* narrative review, *SR* systematic review

Articles are ordered according to their date of publication. The date of the last literature search was extracted from the paper

### Findings of the reviews included

The aim and the main conclusions of the nine reviews were schematically reported in the Table 2. Briefly, the reviews identified several potential thyroid consequences in COVID-19 patients, while no relevant data were found regarding the potential impact of COVID-19 on the clinical/therapeutic management of patients with thyroid diseases. Patients with SARS-CoV-2 infection can develop thyrotoxicosis or low-T3 syndrome and should be at risk to develop subacute thyroiditis (SAT). Regarding the rate of these complications/dysfunctions, no significant data were found in the reviews included in the present systematic review. Furthermore, having a thyroid disease should not be considered a risk factor for SARS-CoV-2 infection. The summary of findings of the present systematic review was reported in Table 3 to facilitate the presentation of the results.

**Table 2:** Aim and main conclusions of the reviews included in the present systematic review

First author [ref]	Aim	Main conclusions
Dworakowska [11]	To explore COVID-19 risks in patients with preexisting thyroid problems. To review current literature on thyroid diseases (excluding cancer) and COVID-19, including data from the previous coronavirus pandemic caused by the SARS-CoV	There are no data suggesting that thyroid patients are at higher risk of COVID-19. In patients severely affected by COVID-19, changes in thyroid function may relate to a ‘sick euthyroid’ syndrome, but there may be specific thyroid-related damage which requires further investigation
Gorini [12]	To summarize the main findings on thyroid and COVID-19 and define research lines aimed at patient care and effective public health measures	Thyroid disease is not a risk factor for the development of COVID-19, and a higher prevalence of thyroid disease has not been found in patients with COVID-19. Questioning patients about the presence of thyroid disease at the time of hospitalization with subsequent follow-up might be useful in patients with multiple diseases to avoid thyrotoxicosis first and hypothyroidism later resulting from SAT
Caron [13]	To discuss the diagnosis and the management of patients presenting with thyrotoxicosis, thyroid-associated orbitopathy and hypothyroidism in the context of SARS-CoV-2 infection	Routine assessment of thyroid function in the acute phase for COVID-19 patients requiring intensive care is useful, as they frequently present thyrotoxicosis related to SARS-CoV-2, and during convalescence to diagnose and adapt levothyroxine replacement treatment in patients with primary or central hypothyroidism
Scappaticcio [7]	To explore the impact of COVID-19 on the thyroid gland	SAT mainly occurs during or soon after mild COVID-19. Thyrotoxicosis without neck pain (possibly in the context of the nonthyroidal illness syndrome) could characterize more severe and critical cases of COVID-19 pneumonia. Some clues of the hormonal changes (i.e., low T3 and TSH concentrations) and overt thyrotoxicosis to be regarded as predictors of poor outcome of COVID-19 are already emerging
Kumari [14]	To explore the potential role of prevailing thyroid disorders in SARS-CoV-2 infection	Thyroid dysfunction may have considerable risk in aggravating the infection and spread of SARS-CoV-2, and it is closely associated with aging
Chen [15]	To review the potential interaction between COVID-19 and the thyroid gland, including thyroid pathology, function and diseases. To explore the potential harmful effects of COVID-19 drugs on the thyroid	SAT caused by SARS-CoV-2 has been reported. The alteration of thyroid tests is common among COVID-19 patients. However, there is no pathological evidence of thyroid injury caused by SARS-CoV-2. Some anti–COVID-19 agents may cause thyroid injury or affect its function
Speer [16]	To explore and compare the impact of SARS-CoV and SARS-CoV-2 on the thyroid gland	No increase of prevalence of pre-existing thyroid disorder in SARS-CoV and SARS-CoV-2 patients was found. However, routine screening of thyroid function at least in COVID-19 patients requiring hospitalization is suggested, because subacute thyroiditis might be a late complication
Croce [17]	To summarize studies regarding thyroid function alterations in patients with COVID-19	Non-thyroid illness syndrome is the most consistently observed alteration of thyroid function parameters. SARS-CoV-2 may also infect the thyroid producing typical (painful) or atypical (painless) subacute thyroiditis
Piticchio [18]	To discuss the relationship between COVID-19 infection and the endocrine glands and compare it with SARS-CoV	A possible damage of endocrine system in COVID-19 patients should be investigated in both COVID-19 acute phase and recovery to identify both early and late endocrine complications that may be important for patient’s prognosis and well-being after infection

*COVID-19* coronavirus disease 2019, *SARS-CoV-2* severe acute respiratory syndrome coronavirus 2, *SAT* subacute thyroiditis

**Table 3:** Summary of findings of the present systematic review

Question of the present systematic review	Conclusion	References supporting these findings
Which impact on thyroid can be expected in COVID-19 patients?	COVID-19-related thyroid dysfunctions include thyrotoxicosis, hypothyroidism, nonthyroidal illness syndrome. It is difficult to distinguish whether altered thyroid function is a result of direct or indirect effects of viral infection. Low-T3 as well as thyrotoxicosis should be predictors of poor outcome of hospitalized COVID-19 patients. SAT occurrence in patients with COVID-19 has been reported	7, 11, 13, 15, 16, 17, 18
Is there a higher risk for SARS-CoV-2 infection among thyroid patients?	Thyroid disease should not be considered a risk factor for SARS-CoV-2 infection	12, 16
Has the management of thyroid patients to be adapted in presence of SARS-CoV-2 infection?	Data on the indication for monitoring thyroid patients when infected by SARS-CoV-2 are unclear and the need of thyroid follow-up is not supported	12, 14, 15, 16

*COVID-19* coronavirus disease 2019, *SARS-CoV-2* severe acute respiratory syndrome coronavirus 2, *SAT* subacute thyroiditis

## Discussion

With the beginning of COVID-19 pandemic, we faced a tsunami of publications. This is true also for the endocrine and thyroid field. However, after one year since the WHO declaration of pandemic, we should need taking stoke of the situation. We have several clues that SARS-CoV-2 can have effects on the thyroid gland. High levels of expression of ACE2 receptor and transmembrane protease serine 2 have been found in thyroid cells [8, 9], abnormal immune responses and cytokine storm associated to COVID-19 may induce thyroid gland inflammation [9, 19], and both direct and indirect mechanisms might affect hypothalamic-pituitary-thyroid axis [20–24].

Based on the above evidences, and according to the sparse knowledge on this topic, a systematic review was designed and a number of nine reviews focused on the relationship between thyroid and SARS-CoV-2 were retrieved. Unfortunately, among the reviews included seven, were narrative reviews that do not represent an evidence-based information as the systematic ones well. Regarding the first question of the present review, the pooled findings from the included reviews allow us to conclude as follows. A subject diagnosed with COVID-19 can: 1) develop thyroid dysfunction due to direct and indirect damage on the thyroid gland; 2) have a low-T3 syndrome if hospitalized due to severe COVID-19 infection; 3) eventually develop SAT. In the nine reviews we did not find clear data allowing to establish the real prevalence of these scenarios. Concerning the other two questions we raised with this review, we can conclude that having a thyroid disease does not represent a risk for infection of SARS-CoV-2 and does not require specific recommendations.

The COVID-19 pandemic has dramatically influenced our daily life and has abruptly shifted the medical research. Furthermore, we have faced a huge medical information, also in the thyroid field, that was often conflicting with each other [25]. Following these considerations, the results from the present paper should be discussed with caution and with the aim to provide further insights from a clinical point of view. It is recognized that patients with severe systemic disease generally show altered thyroid laboratory tests [26]. Also, it is known that patients in the intensive care unit typically present with decreased tri-iodothyronine, low thyroxine, and normal range or slightly decreased TSH (i.e., non-thyroidal illness syndrome or low-T3 syndrome), even if no proof exists for causality of this association [27]. Furthermore, it is known that thyroid hormones levels can hold a role to predict a worse prognosis in these patients [27]. The larger part of studies investigating thyroid function in COVID-19 patients enrolled critically ill subjects. Then, it should be not surprising that also COVID-19 patients present with this thyroid hormone profile, which should not be specifically ascribed to COVID-19. A certain role for systemic inflammation might also be envisaged. Indeed, the cytokine storm occurring during SARS-CoV-2 infection often results in an uncontrolled inflammatory response that is detrimental to host cells [17, 22]. As far as the thyroid gland is concerned, it should be highlighted that several models, including the chronic low-level inflammation associated with aging, the so-called “inflammaging” [28], and the oxidant/antioxidant imbalance associated with thyroid chronic inflammation [29], proved to have a role in the pathogenesis of thyroid diseases. From this point of view, there is potential to consider a thyroid role in the long-term effects of COVID-19.

In addition, it has to be taken into account that the evaluation of thyroid function in COVID-19 patients can be influenced by a number of the medications received [30]. Heparin has been largely used in COVID-19 patients and it is known to interfere in free thyroid hormones assays. In fact, heparin liberates lipoprotein lipase from the vascular endothelium, and blood samples from heparin-treated patients have increased lipoprotein lipase activity generating non-esterified fatty acids (NEFA). Free thyroid hormone assays, especially those with prolonged incubation periods, are affected since NEFA displace T4 and T3 from binding proteins, causing spuriously high values [31]. Patients with severe COVID-19 can develop a systemic inflammatory response and corticosteroids have been largely used because of their powerful anti-inflammatory effects. However, corticosteroids can influence thyroid laboratory tests by several pathways: act on hypothalamic-pituitary control on the thyroid reducing TSH, reduce levels of thyroxine-binding globulin and increase free T4, and inhibit thyroid hormone activation (i.e., conversion from T4 to T3). Finally, it cannot be excluded that COVID-19 patients have been exposed to multiple chest iodinated contrast-enhanced computed tomography which can obviously determines a transient thyrotoxicosis.

Some considerations on the possible occurrence of SAT in patients experiencing COVID-19 should be drawn. Indeed, these descriptions mainly rely on case reports [7]. As a consequence, there is no definite proof about this possible COVID-19 complication, and the true frequency of SAT among COVID-19 is unknown. SAT might occur a few weeks after a viral infection of the upper respiratory tract, and then it might be a late complication of COVID-19. Here we systematically reviewed all reviews published on the relationship between thyroid and COVID-19. The nine reviews we included in our study were published between June 2020 and February 2021 and more recent original studies were not included. Then, some conclusions we achieved might be changed. Regarding the possible need to adapt the management of thyroid patients when infected by SARS-CoV-2, even if we did not find strong information, it has to be cited the recent bulletin by European Thyroid Association [32] quoting that no consequences have to be expected in patients receiving treatment for hypo- or hyperthyroidism [33, 34].

The present systematic review has some limitations to be disclosed. First, published data on the relationship between thyroid and SARS-CoV-2 are limited and sparse. Second, we found almost only narrative reviews that do not represent an evidence-based information. Third, the COVID-19 pandemic is still going on and data we found in the reviews should be revised in the next studies.

In conclusion, the present systematic review of reviews found that: 1) patients diagnosed with COVID-19 can develop thyroid dysfunction, frequently non-thyroidal illness syndrome when hospitalized in intensive care unit, 2) having a thyroid disease does not increase the risk for SARS-CoV-2 infection, 3) thyroid patients do not need a COVID-19-adapted follow-up. Anyway, it has to be taken into account that several COVID-19 intrinsic factors, such as critical illness and medications, could affect thyroid laboratory tests. Since the rapid worldwide diffusion of SARS-CoV-2 and its variants, it is highly important to annotate that the summary of findings of the present study might change in the next future.

## Abbreviations

ACE-2: Angiotensin-converting enzyme 2
COVID-19: Coronavirus disease 2019
NEFA: Non-esterified fatty acids
SARS-CoV-2: Severe acute respiratory syndrome coronavirus 2
SAT: Subacute thyroiditis
